# Robust Virome Profiling and Whole Genome Reconstruction of Viruses and Viroids Enabled by Use of Available mRNA and sRNA-Seq Datasets in Grapevine (*Vitis vinifera* L.)

**DOI:** 10.3389/fmicb.2020.01232

**Published:** 2020-06-05

**Authors:** V. Kavi Sidharthan, Amitha Mithra Sevanthi, Sarika Jaiswal, V. K. Baranwal

**Affiliations:** ^1^Division of Plant Pathology, Indian Council of Agricultural Research-Indian Agricultural Research Institute, New Delhi, India; ^2^Indian Council of Agricultural Research-National Institute for Plant Biotechnology, New Delhi, India; ^3^Centre for Agricultural Bioinformatics, Indian Council of Agricultural Research-Indian Agricultural Statistics Research Institute, New Delhi, India

**Keywords:** virome, mRNAome, sRNAome, grapevine, India

## Abstract

Next-generation sequencing (NGS) based virome analyses of mRNA and sRNA have recently become a routine approach for reliable detection of plant viruses and viroids. In the present study we identified the viral/viroidal spectrum of several Indian grapevine cultivars and reconstructed their whole genomes using the publically available mRNAome and sRNAome datasets. Twenty three viruses and viroids (including two variants of grapevine leafroll associated virus 4) were identified from two tissues (fruit peels and young leaves) of three cultivars among which nine unique grapevine viruses and viroids were identified for the first time in India. Irrespective of the assemblers and tissues used, the mRNA based approach identified more acellular pathogens than the sRNA based approach across cultivars. Further, the mRNAome was on par with the whole transcriptome in viral identification. Through *de novo* assembly of transcriptomes followed by mapping against reference genome, we reconstructed 19 complete/near complete genomes of identified viruses and viroids. The reconstructed viral genomes included four larger RNA genomes (>13 kb), a DNA genome (RG grapevine geminivirus A), a divergent genome (RG grapevine virus B) and a genome for which no reference is available (RG grapevine virus L). A large number of SNPs detected in this study ascertained the quasispecies nature of viruses. Detection of three recombination events and phylogenetic analyses using reconstructed genomes suggested the possible introduction of viruses and viroids into India from several continents through the planting material. The whole genome sequences generated in this study can serve as a resource for reliable indexing of grapevine viruses and viroids in quarantine stations and certification programs.

## Introduction

Grapevine (*Vitis vinifera* L.) is an important cash crop grown worldwide (McGovern, 2003). Being a clonally propagated crop, grapevine is amenable for coinfection by different viruses and viroids (Jo et al., 2018). It is reported to be susceptible to the largest number of acellular pathogens compared to other crop species (Beuve et al., 2018; Hily et al., 2018). Till date, more than 70 viruses and 7 viroids have been reported to infect grapevine (Singhal et al., 2019). Many a time, grapevine viruses deviate from the classical ‘one pathogen – one disease’ concept, i.e., interaction among more than one viral agent leads to disease development (Byrd and Segre, 2016).

Planting pathogen free crop propagules is of paramount importance in grapevine for increasing the productive life of vineyards (Kumar et al., 2015). Traditional detection techniques like ELISA, PCR and their variants are employed for indexing of a few selected viruses of grapevine while certifying the planting material for commercial planting. But these methods can only answer whether the pathogen(s) under investigation is present or not, leaving the status of all other untested viruses in the planting material unknown (Czotter et al., 2018). To assure the health of the planting material of grapevine that remains productive in the field for an average of 15 years in India, it would be necessary to subject it to rigorous indexing for all possible grapevine infecting viruses/viroids. For this, it would be essential to study the virome (total viral population) of the mother stock, the results of which can then be used for developing appropriate detection assays of all pathogens for screening the clonal propagules. Next-generation sequencing (NGS) approaches can provide us with a snapshot of the virome present in the propagule as they are effective not only in detecting the known viral pathogens and their variants, if any, but also in unravelling unknown one(s) (Jo et al., 2018). Among the various NGS based approaches, sRNA (sRNAome) and mRNA (mRNAome) sequencing are commonly used to reveal the virome of a given sample (Pantaleo et al., 2010; Pirovano et al., 2015; Jones et al., 2017; Maliogka et al., 2018; Pooggin, 2018; Massart et al., 2019). Recently, a few studies attempted to reveal the virome of different crops like grapevine, apple, and pepper from publically available mRNAome data (Jo et al., 2015, 2016, 2017).

Both sRNA and mRNA pools can effectively capture single as well as double strand RNA viruses and some DNA viruses (Seguin et al., 2014; Roossinck et al., 2015; Jo et al., 2017). However, the relatively lower representation of viral RNA in the background of total plant RNA limits the use of mRNAome compared to sRNAome for viral detection (Beuve et al., 2018; Maliogka et al., 2018). As mRNA based methods can give longer contigs, they are more useful for variant detection, especially when significant genetic diversity exists as found in some of the grapevine viruses such as grapevine leafroll associated virus 3 (GLRaV3) (Xiao et al., 2019). Thus, it would be worthwhile to study the virome using both these methods for robust identification of entire virome of a plant species.

Though India grows grapevine on 137,000 hectares and exports 185,172 tonnes of grapes annually (FAOSTAT, 2017), only a few studies have been attempted to detect grapevine viruses in India. All these studies targeted only one/few virus(es)/ viroid(s) at a time using traditional detection methods (Kumar et al., 2012, 2013; Sahana et al., 2013; Adkar-Purushothama et al., 2014; Rai et al., 2017; Marwal et al., 2019; Singhal et al., 2019). The current study is the first virome report of grapevines from India using sRNA and mRNA datasets of three Indian grapevine cultivars available in the public domain (Tirumalai et al., 2019) identifying a large number of viruses and viroids.

## Materials and Methods

### Plant Materials and Library Construction

**FIGURE 1:**
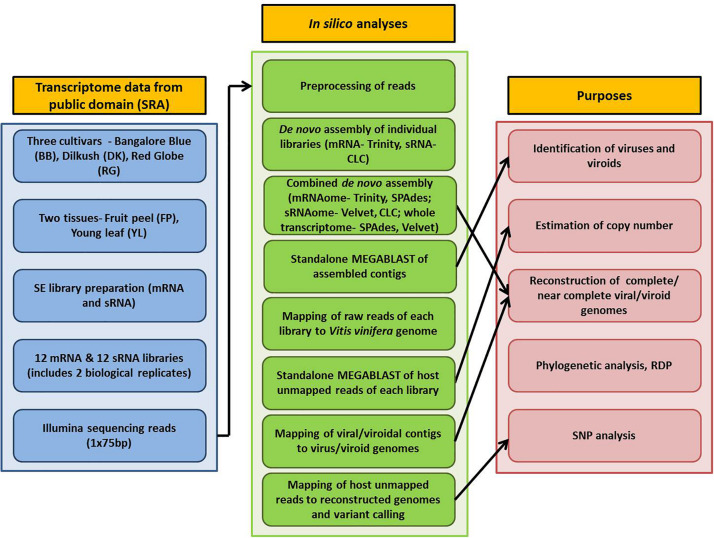
Schematic representation of the viromic study of three Indian grapevine cultivars.

Detailed information on plant materials and library construction is available in Tirumalai et al. (2019). In brief, total RNA was isolated from fruit peels (FP) and young leaves (YL) of three grapevine cultivars- Bangalore Blue (BB), Dilkush (DK), and Red Globe (RG). mRNA-seq and sRNA-seq libraries with two biological replicates, 24 in total, were constructed from isolated total RNA according to the NEXT flex Rapid directional mRNA-seq bundle library protocol (Trapnell et al., 2012) and the TruSeq Small RNA Sample Preparation Guide (Illumina, San Diego, CA, United States) respectively. Sequencing was performed on the Illumina NextSeq500 platform which yielded 75 bp single end reads. Thus, a total, of 12 mRNA and 12 sRNA libraries obtained from two tissues (FP, YL) of three grapevine cultivars (BB, DK, RG) in two biological replicates were analyzed in the current study. The details of the materials used and the complete processing pipeline are indicated in Figure 1.

### Raw Data Pre-processing and *de novo* Assembly of Pre-processed Reads

The bioinformatics analyses were performed using Advanced Super Computing Hub for Omics Knowledge in Agriculture (ASHOKA) facility at ICAR-IASRI, New Delhi, India. Raw data of 24 libraries were downloaded from SRA database and converted to FASTQ files using the SRA toolkit version 2.9.6 (Leinonen et al., 2010). Three approaches were followed for *de novo* assembly. In the first approach, mRNA and sRNA libraries, 12 each, were individually assembled using Trinity version 2.5.1 (Grabherr et al., 2011) and CLC genomics workbench 12 *de novo* assembly tool, respectively with default parameters. For the second approach, combined mRNAome or sRNAome for each cultivar was obtained by aggregating corresponding mRNA or sRNA reads, respectively from four libraries (including two tissues and two replicates) of each individual cultivar. Similarly, whole transcriptome of each cultivar was obtained by aggregating both mRNA and sRNA reads from eight libraries of individual cultivar in the third approach. Trinity (*k* = 25), SPAdes (*k* = 21,23,25) version 3.13.1 (Bushmanova et al., 2019) and CLC (automatic word size = 20), Velvet (*k* = 17) version 1.2.10 (Zerbino and Birney, 2008) were used to assemble combined mRNAomes and sRNAomes, respectively while whole transcriptomes were assembled using SPAdes (*k* = 17,19,21) and Velvet (*k* = 21) assemblers.

### Identification of Viruses and Viroids and Copy Number Estimation

All the assembled contigs were subjected to standalone MEGABLAST analysis (e-value cut off: 1e-5; query coverage: ≥ 80%) against the complete reference sequences of viruses and viroids^1^ using NCBI blast+ version 2.9.0. Only contigs of greater than 50 (for sRNAome) and 200 nucleotides (mRNAome and whole transcriptome) were considered for analyses. To validate the viruses/viroids identified through assembly, the reads of each mRNA/sRNA library were first mapped to the *Vitis vinifera* genome (GCF_000003745.3) using CLC workbench mapping tool with default parameters (match score-1, mismatch cost-2, length fraction-0.5, similarity fraction-0.8). The unmapped reads were then analyzed using MEGABLAST algorithm (e-value cut off: 1e-5; query coverage: ≥80%) against the reference genomes of viruses and viroids. Only those viruses/vioids that were detected through assembly (from sRNAome/mRNAome/whole transcriptome) and BLAST analysis of reads from at least two libraries (derived from the particular nucleic acid pool from which the contigs were obtained) of the corresponding cultivar were considered. To arrive at the copy number for a virus/viroid, the number of reads associated with either RdRp ORF (in case of viruses that use sub-genomic RNA (sgRNA) for translation) or the entire polyprotein [in grapevine fleck virus (GFkV), grapevine rupestris vein feathering virus (GRVFV)] or the entire genome (in viroids) was multiplied with 75 (for mRNA)/ 24 (for sRNA) followed by division with the size (bp) of the corresponding genomic region of the virus/viroid. Intact mRNA reads were used for copy number estimation while the pre-processed reads were used in case of sRNA. The average length of pre-processed sRNA reads in all libraries was near to 24. Hence the factors 75 and 24 were used for mRNA and sRNA libraries, respectively. As a reference genome for grapevine virus L (GVL) was not available in NCBI, we included the *de novo* assembled GVL genome of the present study (that was identified by performing BLASTn analysis of larger contigs against “non-redundant” (nr) (NCBI) database) for MEGABLAST analysis.

### Reconstruction of Whole Genomes of Viruses and Viroids

Virus/viroid associated contigs were filtered from the total contigs using SAM tools version 1.9 (Li et al., 2009). The detailed procedure followed for genome reconstruction is given in Supplementary Figure S1. In brief, the Trinity assembled longer contigs from combined mRNAomes were examined for the presence of intact viral/viroidal genome. Further, the SPAdes assembled longer contigs from combined mRNAomes and whole transcriptomes were examined followed by inspection of Trinity assembled larger contigs in individual mRNA libraries. Next, the Trinity assembled viral/viroidal contigs from combined mRNAomes were mapped against the NCBI designated reference genomes of identified viruses and viroids (CLC workbench mapping tool). In cases where the Trinity assembled contigs were insufficient to reconstruct the entire genome, SPAdes assembled contigs from combined mRNAomes and whole transcriptomes were supplemented during mapping. Still, if the genome could not be obtained, the most closely related genome was used as reference during mapping. The full length consensus sequence, if obtained, after mapping/directly by *de novo* assembly was considered as the complete/near complete genome for a particular virus/viroid. To find ORFs in assembled viral genomes, we used NCBI ORF finder^2^.

### Pairwise Distance and Phylogenetic Analyses

The complete genomes retrieved from NCBI along with the viral/viroid genomes reconstructed in this study were aligned using CLUSTALW tool in MEGA7 software version 7.0.26 (Kumar et al., 2016). Aligned sequences were subjected to pairwise distance analysis and phylogenetic tree construction using neighborhood joining (NJ) method and Kimura 2-parameter (K2P) model with 1000 bootstrap replicates. For grapevine geminivirus A (GGVA), grapevine latent viroid (GLVd), grapevine leafroll associated virus 4 (GLRaV4), grapevine virus B (GVB), GVL, grapevine rootstock stem lesion associated virus (GRSLaV) and GRVFV, all the respective complete genomes available in NCBI were used for analysis. Owing to the availability of a large number of genome sequences for GLRaV3, only those sequences showing 100% query coverage in BLASTn analysis against nr (NCBI) database were taken for analysis. Similarly, in cases of Australian grapevine viroid (AGVd), grapevine yellow speckle viroid-1, -2 (GYSVd1, GYSVd2), and hop stunt viroid (HSVd), only 10 non-redundant genomes that were highly similar to each isolate of a viroid were used. In all the cases, an outgroup (except for pairwise distance analysis) and the NCBI designated reference genome (except GVL, for which there is no designated reference sequence) were included.

### Single Nucleotide Polymporphism (SNP) Analyses

The host unmapped reads of individual cultivars were mapped against the complete/near complete viral/viroid genomes assembled from the corresponding cultivar using the mapping tool available in CLC workbench using default parameters (match score-1, mismatch cost-2, length fraction-0.5, similarity fraction-0.8). For SNP detection, the mapped files were subjected to fixed ploidy variant detection using CLC workbench. As viral genomes are haploid, ploidy value was considered as one throughout the analyses.

### Recombination Analyses

Using CLUSTALW aligned MEGA file as input, recombination analysis was performed using RDP4 package version 4.39 (Martin et al., 2015) employing nine different algorithms. Only recombination events detected by at least five algorithms in the reconstructed viral genomes were considered. Only viral sequences used for phylogenetic analyses were used for detection of recombinants.

## Results

### Pre-processing of Raw Data

The number of raw reads ranged from 10.5 to 40.2 million with an average of 23.3 million for mRNA and 2.9 to 8.3 million with an average of 4.3 million for sRNA libraries (Table 1). As mRNA reads were of acceptable quality (without adapter sequences; phred-score > 20), we proceeded directly for *de novo* assembly while sRNA reads were filtered to remove adapter sequences and poor quality reads (quality scores < 0.05).

**TABLE 1:** Raw reads, host unmapped reads, and viral/viroid associated reads of each library.

Library	Raw mRNA reads	Raw sRNA reads	Host unmapped mRNA reads	Host unmapped sRNA reads	Viral/viroidal mRNA reads	Viral/viroidal sRNA reads	Proportion of host unmapped reads from mRNAome	Proportion of host unmapped reads from sRNAome	Proportion of viral/viroid reads as the percentage of unmapped mRNA reads^a^	Proportion of viral/viroid reads as the percentage of unmapped sRNA reads^a^
BBFPR1	10544229	3726821	449774	389040	8699	11	4.27	10.44	1.93	0.003
BBFPR2	23438185	5250400	989202	515220	19392	17	4.22	9.81	1.96	0.003
BBYLR1	13569098	3115864	414503	274662	1477	2	3.05	8.81	0.36	0.001
BBYLR2	40239577	4317810	1225095	284457	4180	11	3.04	6.59	0.34	0.004
DKFPR1	25945875	3789502	840303	430384	19303	113	3.24	11.36	2.30	0.026
DKFPR2	23865863	8263827	781885	697282	16383	413	3.28	8.44	2.10	0.059
DKYLR1	23773201	2962599	899366	179017	5003	11	3.78	6.04	0.56	0.006
DKYLR2	26500597	5197911	1003686	246769	5467	44	3.79	4.75	0.54	0.018
RGFPR1	22622837	4936802	1803089	689055	78225	73	7.97	13.96	4.34	0.011
RGFPR2	21927732	3251448	1795168	599065	73229	19	8.19	18.42	4.08	0.003
RGYLR1	26815464	2942096	892189	253236	26128	175	3.33	8.61	2.93	0.069
RGYLR2	20471622	3421076	643008	336020	21035	126	3.14	9.82	3.27	0.037
Average	23309523.3	4264679.67	978105.67	407850.58	23210.08	84.58	4.27	9.75	2.06	0.020

*^*a*^Only reads with qcov ≥ 80 were considered.*

### Identification of Viruses and Viroids From Grapevine mRNAome and sRNAome

**FIGURE 2:**
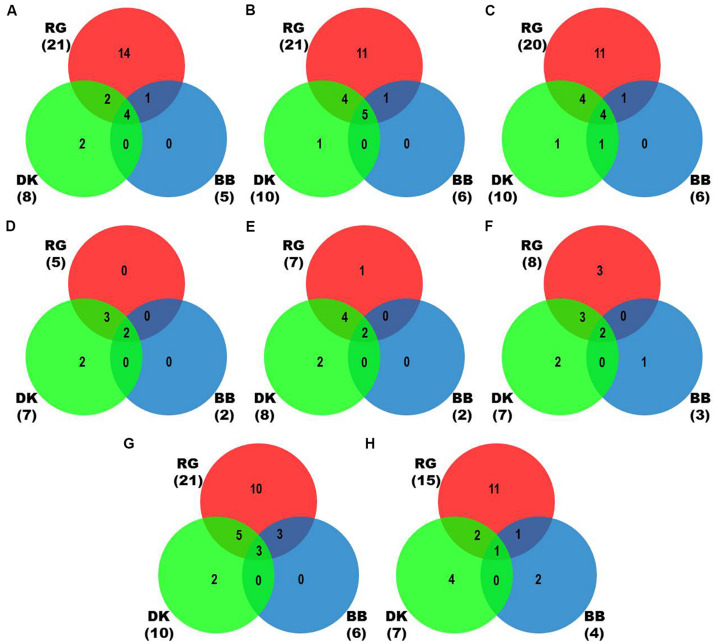
Identification of viruses and viroids in three Indian grapevine cultivars – BB, DK, RG. Venn diagrams display the number of identified viruses and viroids based on Trinity assembled contigs from individual mRNA libraries of each cultivar **(A)**, Trinity **(B)**, SPAdes (*k* = 21,23,25) assembled contigs from combined mRNAome of each cultivar **(C)**, CLC assembled contigs from individual sRNA libraries of each cultivar **(D)**, CLC **(E)**, Velvet (*k* = 13,15,17) assembled contigs from combined sRNAome of each cultivar **(F)**, SPAdes (*k* = 17,19,21) **(G)** and Velvet (*k* = 17,19,21) **(H)** assembled contigs from whole transcriptome of each cultivar. Blue, green and red circles in the Venn diagrams represent the cvs. BB, DK, and RG, respectively.

**FIGURE 3:**
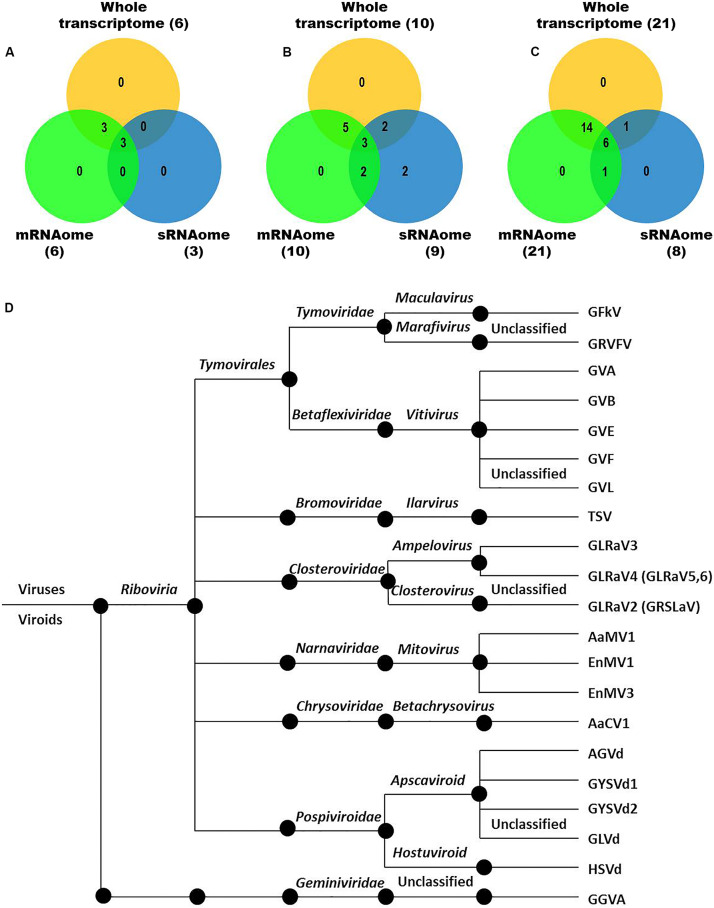
Viruses and viroids identified in three Indian grapevine cultivars – BB, DK, RG. Venn diagram displaying viruses and viroids identified by any of the assemblers from combined mRNAomes, sRNAomes and whole transcriptomes in cultivars BB **(A**), DK **(B),** and RG **(C)**. Blue, green, and yellow circles in the Venn diagrams represent the sRNAome, mRNAome, and whole transcriptome, respectively. Classification of the identified viruses and viroids is on the basis of taxonomy **(D)**.

We identified more viruses and viroids from mRNAome (23) than sRNAome (7) across cultivars and tissues (Supplementary Table S1). The only exception for this was the FP-specific sRNA datasets of cv. DK which identified six viruses and viroids while the corresponding mRNAome could identify only five. Among the two tissues, relatively more viruses/viroids were identified in FP than YL in all cultivars except DK from mRNA libraries. However, nearly similar number of viruses/viroids was identified from sRNA libraries across tissues and cultivars (Supplementary Figure S2 and Supplementary Table S1). Combined mRNAome assembly using Trinity identified the same number of viruses and viroids (23) across cultivars as compared to the individual mRNA libraries (23). However, on a closer look, we found that combining the reads of the two tissues of each cultivar did offer some advantage in case of mRNAome, since additional virus(es)/viroid(s) were identified in BB (1), DK (2) and RG (1). The only exception to this is GVE that was detected in individual mRNA libraries but not in combined mRNAome (Figures 2A,B, Supplementary Figures S3A,B, and Supplementary Table S2). Similarly, combined sRNAome assembly using CLC was more effective as it could identify two unique viruses (GVF in DK and GRSLaV in RG) in addition to the seven viruses and viroids identified by the individual library approach across cultivars (Figures 2D,E, Supplementary Figures S4A,B, and Supplementary Table S3). Between the combined sRNAome and combined mRNAome, the former could identify only a fraction of viruses and viroids (12) identified by the latter even after accounting for the viruses and viroids identified by all the assemblers. Interestingly, from combined mRNAomes and whole transcriptomes exactly the same number of viruses/viroids was identified in BB, DK and RG cultivars (6, 10, and 21), representing a total of 23 viruses/viroids though the identities of a few differed in cvs. DK and RG. The identified acellular pathogens included 14 grapevine viruses (including two GLRaV4 variants), four mitoviruses and five viroids – Alternaria alternata chrysovirus 1 (AaCV1), Alternaria arborescens mitovirus 1 (AaMV1), AGVd, Erysiphe necator mitovirus 1 (EnMV1), Erysiphe necator mitovirus 3 (EnMV3), GFkV, GGVA, GLVd, GLRaV3, grapevine leafroll associated virus -4, -5, -6 (GLRaV4, GLRaV5, GLRaV6), GVA, GVB, GVL, GVE, GVF, GRSLaV, GRVFV, GYSVd1, GYSVd2, HSVd and tobacco streak virus (TSV). It is worthy of mention that while TSV was detected from cvs. BB, RG in the mRNAome, none of the sRNA libraries could detect it (Supplementary Tables S2, S3). Interestingly, GLRaV3, GVA, GVB, GYSVd1, and HSVd were identified in all the cultivars (Figures 3A–D and Supplementary Table S4).

### Performance of Different Assemblers in Identification of Viruses and Viroids From mRNAome, sRNAome, and Whole Transcriptomes

In case of combined mRNAomes, the number of viruses and viroids identified by both the assemblers were similar except in cv. RG where Trinity identified one additional viroid (GYSVd1) compared to SPAdes (Figures 2B,C and Supplementary Table S2). However, both the assemblers identified almost similar number of viral contigs for all but one cultivar (Supplementary Figures S3B,C). In case of combined sRNAomes, Velvet identified one additional virus in BB (GVA) and RG (GVE) than CLC but CLC detected two Velvet undetected virus/viroid in DK (GVF, GYSVd1). Interestingly, GVB was identified in cv. DK by Velvet but not by CLC (Figures 2E,F and Supplementary Table S3). Considering the number of viral and viroid contigs, Velvet identified 67.3, 14.7, and 52.3% more contigs than CLC in BB, DK, and RG (Supplementary Figures S4B,C). In case of whole transcriptome assembly, SPAdes identified more viruses (2, 3, and 6 additional viruses/viroids in cvs. BB, DK, and RG, respectively) and viral contigs as compared to Velvet in all cultivars. Notably Velvet based assembly failed to identify HSVd from any whole transcriptome, or GYSVd2 from DK or GYSVd1,2 from RG, whereas SPAdes identified HSVd in BB and RG, and GYSVd2 in DK and GYSVd1,2 in RG from the corresponding whole transcriptomes. However, Velvet did detect HSVd in each of the combined sRNAomes and GYSVd2 from DK and GYSVd1,2 from RG (Figures 2G,H, Supplementary Figures S5A,B, and Supplementary Tables S3, S5).

### Copy Number Estimation for Identified Viruses and Viroids in Each mRNA and sRNA Library

The number of host unmapped mRNA and sRNA reads ranged from 0.41 to 1.80M and 0.18 to 0.70M, respectively across libraries. Though the number of host unmapped reads was higher (0.98M) in case of mRNA compared to sRNA (0.41M), the proportion of unmapped reads to total reads was higher in the latter (9.75%) than the former (4.27%). On average, 2.06 and 0.02% of host-unmapped reads from mRNA and sRNA libraries mapped to viral/viroidal genomes (Supplementary Figures S6A,B and Table 1). In general, the proportion of virus/viroid associated reads was relatively higher in mRNA libraries constructed from FP than YL while no such trend was observed in case of sRNA libraries. Based on copy number estimates, HSVd (94–100%) predominated in cv. BB in both mRNA and sRNA libraries. In case of mRNA libraries of cvs. DK and RG, HSVd and GYSVd2 were predominant in FP and YL, respectively. In sRNA libraries of cv. DK and in all but one sRNA libraries of cv. RG, GYSVd2 was predominant irrespective of tissue type (Supplementary Figures S7A,B). Further, both the replicates in each tissue of a cultivar were highly similar not only in detecting the viromes but also in estimating their copy number.

### Viral/Viroid Genome Reconstruction From *de novo* Assembled Contigs

By mapping, the viral/viroid associated contigs from combined mRNAome and whole transcriptome of each cultivar against the NCBI designated reference genomes of identified viruses and viroids we obtained complete or near complete (>99%) genomes of 15 viruses and viroids from three cultivars (Table 2). Some other viral contigs could not be assembled into full genomes using the reference genomes as scaffolds. For the assembly of GLRaV3 and GLRaV4 genomes from cv. RG, the longest Trinity assembled contig of each virus was first blasted against the nr (NCBI) database. The complete genome of the most highly similar isolate was then used as a reference during mapping in each case. Trinity assembly of library RGFPR2 directly yielded the whole genome of GVB. Similarly, we obtained GVL genome from one of the Trinity assembled longest contigs from combined mRNAome of cv. RG through BLAST against nr (NCBI) database. In total, we obtained 19 complete/near complete viral/viroid genomes from three cultivars (Table 2). Trinity yielded relatively longer contigs for most viruses and viroids as compared to SPAdes in all cultivars with mRNA reads (Supplementary Figures S8, S9). On the contrary, SPAdes yielded relatively longer viral/viroid contigs as compared to Velvet in most instances when whole transcriptomes were assembled (Supplementary Figures S10, S11). Though Velvet assembled more viral/viroid contigs from combined sRNAomes, CLC yielded longer contigs for most viruses and viroids as compared to Velvet (Supplementary Figures S12, S13). However, we could not reconstruct any viral genome using contigs assembled from combined sRNAomes. From the reconstructed complete/near complete viral genomes, we could identify all of the anticipated ORFs for all recovered viruses using NCBI ORF finder (Supplementary Table S6). Failure to identify intact ORFs in nearly complete genomes that could be assembled to the tune of >95% (Supplementary Table S7) were still deemed incomplete.

**TABLE 2:** Summary of complete/near complete viral/viroidal genomes reconstructed from three Indian grapevine cultivars.

Sl. no.	Cultivar	Virus/viroid	Reference	Size (nt)	Genome recovery (nt)	Assigned isolate name	GenBank accession number	Contigs assembled from	Library^a^	Reference used for mapping
1	Bangalore Blue	GYSVd1	NC_001920.1	366	367	BB GYSVd1	MN662238	Trinity, SPAdes	CmR, WT	NCBI reference genome
2	Bangalore Blue	HSVd	NC_001351.1	302	300	BB HSVd	MN662243	Trinity, SPAdes	CmR, WT	NCBI reference genome
3	Dilkush	GLRaV3	NC_004667.1	17919	17919	DK GLRaV3	MN662228	Trinity	CmR	NCBI reference genome
4	Dilkush	AGVd	NC_003553.1	369	369	DK AGVd	MN662235	Trinity, SPAdes	CmR, WT	NCBI reference genome
5	Dilkush	GYSVd1	NC_001920.1	366	367	DK GYSVd1	MN662239	Trinity, SPAdes	CmR, WT	NCBI reference genome
6	Dilkush	GYSVd2	NC_003612.1	363	363	DK GYSVd2	MN662241	Trinity, SPAdes	CmR, WT	NCBI reference genome
7	Dilkush	HSVd	NC_001351.1	302	301	DK HSVd	MN662244	Trinity, SPAdes	CmR, WT	NCBI reference genome
8	Red Globe	GLRaV3	MH814489.1	18558	18469	RG GLRaV3	MN662229	Trinity	CmR	Closely related genome*
9	Red Globe	GRSLaV	NC_004724.1	16527	16519	RG GRSLaV	MN662231	Trinity	CmR	NCBI reference genome
10	Red Globe	GLRaV4	KY821095.1	13857	13780	RG GLRaV4	MN662230	Trinity, SPAdes	CmR	Closely related genome*
11	Red Globe	GGVA	NC_031340.1	2905	2905	RG GGVA	MN661401	Trinity	CmR	NCBI reference genome
12	Red Globe	GVB	*De novo*	NA	7621	RG GVB	MN662233	Trinity	RGFPR2	NA
13	Red Globe	GVL	*De novo*	NA	7588	RG GVL	MN662234	Trinity	CmR	NA
14	Red Globe	GRVFV	NC_034205.1	6730	6703	RG GRVFV	MN662232	Trinity	CmR	NCBI reference genome
15	Red Globe	AGVd	NC_003553.1	369	370	RG AGVd	MN662236	Trinity	CmR	NCBI reference genome
16	Red Globe	GLVd	NC_028131.1	328	329	RG GLVd	MN662237	Trinity, SPAdes	CmR, WT	NCBI reference genome
17	Red Globe	GYSVd1	NC_001920.1	366	367	RG GYSVd1	MN662240	Trinity, SPAdes	CmR, WT	NCBI reference genome
18	Red Globe	GYSVd2	NC_003612.1	363	363	RG GYSVd2	MN662242	Trinity	CmR	NCBI reference genome
19	Red Globe	HSVd	NC_001351.1	302	301	RG HSVd	MN662245	Trinity, SPAdes	CmR, WT	NCBI reference genome

^*a*^CmR- Combined mRNAome, WT- Whole Transcriptome, RGFPR2- Red Globe Fruit Peel Replicate 2 (one of the individual libraries). NA- Not Applicable. *Initially RG GLRaV3, 4 genomes could not be reconstructed by using their corresponding NCBI designated reference genomes during mapping. However, use of their most closely related genome, that were identified by BLASTn analysis of longest Trinity assembled contigs against nr (NCBI) database, yielded their near complete genomes.

### Pairwise Distance and Phylogenetic Analyses Using Reconstructed Viral/Viroid Genomes

**FIGURE 4:**
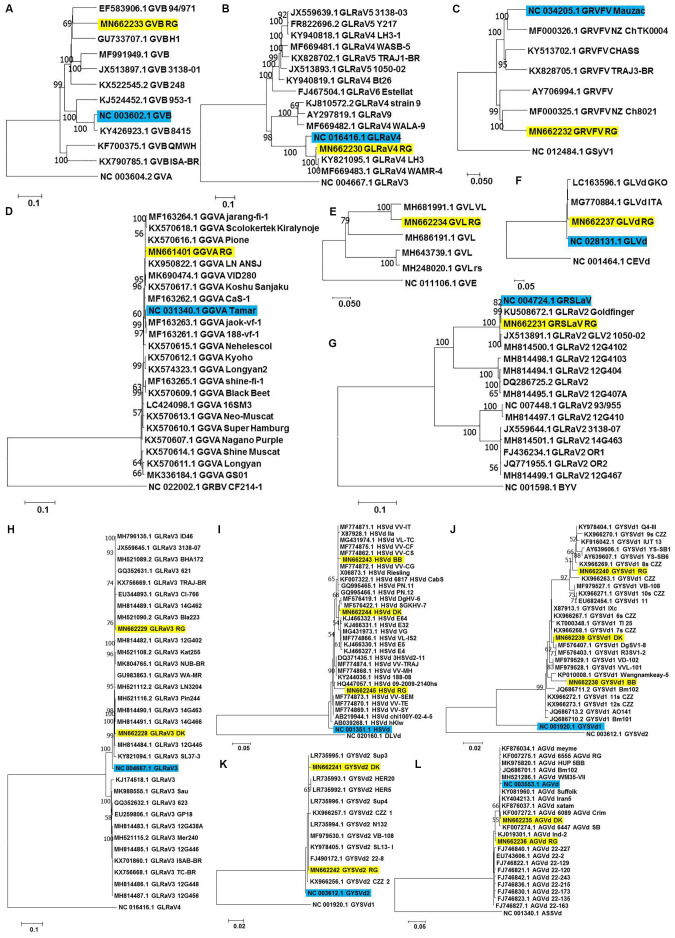
Phylogenetic analyses for genome reconstructed viruses and viroids. Phylogenetic relationships of GVB **(A)**, GLRaV4 **(B)**, GRVFV **(C)**, GGVA **(D)**, GVL **(E)** GLVd **(F)**, GRSLaV **(G)**, GLRaV3 **(H)**, HSVd **(I)**, GYSVd1 **(J)**, GYSVd2 **(K),** and AGVd **(L)** isolates obtained in this study with global isolates. Phylogenetic tree was constructed using Neighborhood joining (NJ) method and Kimura 2-parameter model (K2P) with 1000 bootstrap replicates. Yellow colored boxes indicate the isolates obtained in this study. Blue colored boxes indicate the corresponding NCBI designated reference genome. Only bootstrap values more than 50 are shown. An outgroup was used in the analysis of each virus/viroid.

Each of the complete/near complete genomes obtained were subjected to pairwise distance (Supplementary Tables S8–S19) and phylogenetic analyses (Figures 4A–L) along with related complete genomes retrieved from NCBI, and the most closely related genomes are indicated here, including their country of origin.

#### Viruses

a-RG GVB (MN662233) was related to a South African isolate GVB-H1 [79.7% nucleotide (nt) identity; GU733707.1] (Figure 4A and Supplementary Table S8).
b-RG GLRaV4 (MN662230) was closely related to a Pakistani isolate LH3 (97.9% nt identity; KY821095.1) (Figure 4B and Supplementary Table S9).
c-RG GRVFV (MN662232) was related to a New Zealand isolate NZ Ch8021 (80.6% nt identity; MF000325.1) (Figure 4C and Supplementary Table S10).
d-RG GGVA (MN661401) was most closely related to a Japanese isolate Pione (99.6% nt identity; KX570616.1) (Figure 4D and Supplementary Table S11).
e-RG GVL (MN662234) was closely related to a Croatian isolate VL (94.0% nt identity; MH681991.1) (Figure 4E and Supplementary Table S12).
f-RG GLVd (MN662237) was closely related to an Italian isolate ITA (98.4% nt identity; MG770884.1) (Figure 4F and Supplementary Table S13).
g-RG GRSLaV (MN662231) was most closely related to a Californian isolate obtained from the cv. RG (99.8% nt identity; NC_004724.1) (Figure 4G and Supplementary Table S14).
h-DK GLRaV3 (MN662228) shared 99.0% nt identity with Canadian isolates- 14G463 (MH814490.1), 14G466 (MH814491.1), 14G462 (MH814489.1), 3138-07 (JX559645.1), a Brazilian isolate TRAJ-BR (KX756669.1) and an US isolate WA-MR (GU983863.1).
i-RG GLRaV3 (MN662229) shared 99.4% nt identity with US isolates- Bla223 (MH521090.2), WA-MR 314 (GU983863.1), Canadian isolates- 14G463 (MH814490.1), 14G462 (MH814489.1) and a Brazilian isolate TRAJ-BR (KX756669.1).
j-GLRaV3 isolates from cvs. DK and RG showed only 1.3% divergence (Figure 4H and Supplementary Table S15).

#### Viroids

k-BB HSVd (MN662243) shared 99.3% nt identity with Brazilian isolates- VL-TC (MG431974.1), VV-CF (MF774875.1), VV-CG (MF774872.1), VV-IT (MF774871.1), VV-CS (MF774862.1) and a German isolate obtained from cv. Riesling (X06873.1).
l-DK HSVd (MN662244) was closely related to a Nigerian isolate DgHV-6 (99.3% nt identity; MF576419.1) while RG HSVd (MN662245) was closely related to a New Zealand isolate 09-2009-2140hs (96.8% nt identity; HQ447057.1). Interestingly, RG HSVd diverged largely from DK HSVd (4.7%) and BB HSVd (5.5%) while DK HSVd showed 2.9% divergence from BB HSVd (Figure 4I and Supplementary Table S16).
m-Similarly, while BB GYSVd1 (MN662238) was closely related to a Thailand isolate Wangnamkeay-5 (98.0% nt identity; KP010008.1), DK GYSVd1 (MN662239) shared 99.7% nt identity with a German isolate IXc (X87913.1), two Nigerian isolates DgSV1-8 (MF576407.1), R3SV1-2 (MF576403.1) and two Chinese isolates clone 6s CZZ (KX966267.1), clone 7s CZZ (KX966268.1) and RG GYSVd1 (MN662240) was most closely related to yet another Pakistani isolate Q4-III (99.1% nt identity; KY978404.1).
n-Like HSVd, RG GYSVd1 diverged from DK (3.2%) and BB GYSVd1 (5.0%) while DK GYSVd1 showed 2.9% divergence from BB GYSVd1 (Figure 4J and Supplementary Table S17).
o-RG GYSVd2 (MN662242) was identical (100% nt identity) to Greek isolates- Sup4 (LR735996.1), Sup3 (LR735995.1), N132 (LR735994.1), a Croatian isolate VB-108 (MF979530.1), a Pakistani isolate SL13- I (KY978405.1) and a Chinese isolate clone 22-8 (FJ490172.1) while the DK GYSVd2 (MN662241) isolate shared 99.7% nt identity with all the six closest relatives of RG GYSVd2 and also with the RG GYSVd2 isolate (Figure 4K and Supplementary Table S18).
p-DK AGVd (MN662235) was identical to a Chilean isolate 6089_AGVd_Crim (100% nt identity; KF007272.1) while RG AGVd (MN662236) shared 99.4% nt identity with an Indian isolate Ind-2 (KJ019301.1) and two Chinese isolates- clone 22-2 (EU743606.1), clone 22-129 (FJ746822.1). It is noteworthy that DK and RG AGVd isolates fell within two separate clades (1.9% divergence) (Figure 4L and Supplementary Table S19).

### SNP Detection and Recombination Analyses in Reconstructed Genomes

**FIGURE 5:**
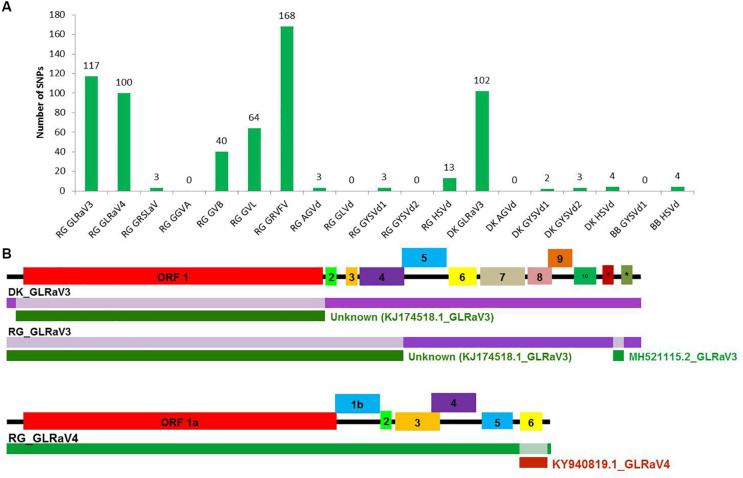
SNP detection and recombination analyses in whole genome reconstructed viruses and viroids. The number of identified SNPs for each virus and viroid **(A)**. Identification of recombination events in reconstructed viral genomes using RDP4 program. **(B)** The positions of identified recombination events are indicated in a different color in the genome. The corresponding minor parental sequence is indicated by a differently colored bar below each event along with the name. #-ORF 11, *-ORF 12 in GLRaV3.

A large number of SNPs was detected for RG GRVFV (168) followed by RG GLRaV3 (117), that were equally distributed throughout the genome, while no SNP was detected in case of RG GGVA, RG GLVd, RG GYSVd2, DK AGVd, and BB GYSVd1. Other viruses that had a good number of SNPs included GLRaV3 from cv. DK (102) and GLRaV4 (100), GVL (64), and GVB (40) from cv. RG (Figure 5A).

The reconstructed viral genomes, after alignment, were subjected to detection of recombination events. Among the eight reconstructed viral genomes, recombination events supported by at least five algorithms were detected in only three genomes. In GLRaV3 genomes of DK and RG, a similar recombination event was detected in 5′ region of the genome. An additional recombination event was detected in 3′ region of RG GLRaV3. For RG GLRaV4, we found only one recombinant sequence at 3′ region (Figure 5B and Supplementary Table S20).

## Discussion

In this study, viromes of three Indian grapevine cultivars were determined and some of their whole genomes were reconstructed from publically available mRNAome and sRNAome datasets (Tirumalai et al., 2019). Since the materials used in the present study were obtained from Indian Institute of Horticultural Research (in Bangalore, India) one of the leading grapevine breeding centers in the tropical region (Tirumalai et al., 2019), it is the most appropriate one for performing virome analysis as all the vegetative propagules derived from the breeding stock would be expected to be infected with the same viruses. Interestingly the cv. RG, an introduction from California had the maximum viral load in our study compared to the native cvs., BB and DK.

Uneven distribution of viruses and viroids across tissues of a perennial plant like grapevine (Kominek et al., 2009), suggested that sampling different tissues will reveal a more accurate sanitary status of a plant. We also found pooling samples from different tissues was more reliable than relying on individual tissue for virome analysis. Earlier, Jo et al. (2015), also reported the superiority of tissues-combined assemblies over the individual ones. We further observed that the combined mRNAome and whole transcriptome identified nearly similar acellular pathogens and both these approaches were more sensitive than individual or combined sRNAomes. This might be because of the smaller size and number of reads generated from sRNA libraries. Contrary to the observation of Maliogka et al. (2018), the proportion of viral and viroidal reads in mRNA libraries was higher than sRNA libraries in our study. This might be due to the fact that viral sRNAs are produced only upon activation of host’s antiviral defense while mRNAomes can even detect viruses and viroids that are unrecognized by the host (Hily et al., 2018). Further, similar number of viruses and viroids were identified by Trinity and SPAdes assemblers from mRNAomes and CLC and Velvet assemblers from sRNAomes. However, SPAdes outperformed Velvet in case of whole transcriptomes. So, when more than one assembler was used, one or more viruses that escaped detection by one assembler could be detected by the other (Massart et al., 2019). Thus, use of multiple tissues and assemblers enabled better unraveling of grapevine virome.

In the present study, we identified 19 grapevine viruses and viroids (including two variants of GLRaV4) and four mycoviruses associated with the grapevine fungal pathogens- *Erysiphe necator* and *Alternaria* spp. (Kakalikova et al., 2009; Feng et al., 2018). Included among these is GRSLaV, which was earlier reported as a novel virus from California in cv. RG. This indicates the possible introduction of GRSLaV from California along with the RG propagule. However, GRSLaV is now regarded as a strain of grapevine leafroll associated virus 2 (GLRaV2) (as GLRaV-2RG) (Alkowni et al., 2011). Nonetheless, this is the first study that could successfully detect GLRaV-2 or any of its variants in India. Though Kumar et al. (2013) did attempt to detect this virus in India they could not succeed rather they detected GLRaV1 and GLRaV3. Further, GLRaV5 and GLRaV6 are presently regarded as the strains of GLRaV4 (Rai et al., 2017). On this basis, nine grapevine viruses and viroids (GGVA, GLRaV2, GRVFV, GVA, GVE, GVF, GVL, TSV, and GLVd) were detected for the first time in grapevine cultivars grown in Indian soil. Interestingly, we could identify GVL, the reference for which is not yet available in the NCBI, using the GVL genome obtained in this study.

Of the 19 complete/near complete genomes (>99% completion but <100%) obtained in this study, seven viral (including four genomes with > 13 kb) and 1 viroidal genome were recovered for the first time from any Indian grapevine cultivar. None of the viral whole genomes could be recovered from combined sRNAome assembled contigs as reported by Baranwal et al. (2015) and Jo et al. (2016). However, this might be due to the use of lower number of sRNA reads (approximately one-fifth) as compared to the mRNA reads in the current study. Identification of DNA viruses in mRNAome is rare and construction of their whole genome is still scarce (Jo et al., 2017), but we could not only identify GGVA in mRNA of cv. RG but could also reconstruct its genome in entirety with 2905 nucleotides. Initially, the RG GVB and GVL genomes could not be recognized as the former diverged significantly (23%) from the reference genome while there was no reference genome for the latter. However, inspection of Trinity assembled longer contigs of individual and combined mRNA libraries through BLAST analysis against nr (NCBI) database coupled with ORF prediction, identified the whole genomes of these isolates. Complete genomes could not be reconstructed for RG GLRaV3, 4 isolates using reference-based mapping because of their divergence (2.7 and 9.6% divergence of RG GLRaV3, 4 genomes, respectively) from the corresponding reference genome, though their near complete genomes could be reconstructed using the closely related genomes. Thus, examination of larger contigs assembled by various *de novo* assemblers coupled with usage of increased number of reference genomes of a virus during mapping could increase the chances of whole genome recovery. Identification of large number of viral SNPs in this study ascertains the quasispecies nature of plant viruses (Jo et al., 2018). Hence, the complete/near complete genomes reconstructed in this study were the consensus of viral variants present in a given cultivar.

We followed Jo et al. (2015) for copy number estimation except that we considered reads from only the non sgRNA region to reflect the true abundance of viruses that use sgRNA strategy for translation. Phylogenetic and distance matrix analyses revealed the divergence of AGVd, HSVd and GYSVd1 isolates obtained from different cultivars while the GLRaV3 and GYSVd2 isolates from cvs. DK and RG were related. Recombination analyses revealed that the RG GLRaV3, DK GLRaV3, and RG GLRaV4 isolates were recombinants of global isolates. Owing to the vegetative propagation of grapevine and free trade of planting materials, viruses and viroids can easily spread globally. In addition, coinfection of a single plant with numerous isolates of same/different viruses offers scope for recombination among different isolates (Jo et al., 2016).

Plants do not always express symptoms associated with every virus/viroid present, hence symptomology and individual virus/viroid based detection assays are not sufficient to determine the full spectrum of viruses/viroids present in a plant. Rather, use of available or newer transcriptome datasets is a better choice for profiling of viromes that can serve as a reliable base for indexing of planting materials in plant quarantine stations and during certification.

## Data Availability Statement

The datasets analyzed in this study are available in the NCBI repository under the Bioprojects PRJNA421907 (sRNA-seq) and PRJNA421908 (mRNA-seq). The whole genomes of 19 viruses and viroids reconstructed in this study have been submitted to GenBank (MN662228 to MN662245 and MN661401).

## Author Contributions

VS, AS, SJ, and VB conceptualized and formulated the study and read and approved the final manuscript. VS performed the bioinformatics analyses. VS and AS have drafted the manuscript. VB and SJ edited the manuscript.

## Conflict of Interest

The authors declare that the research was conducted in the absence of any commercial or financial relationships that could be construed as a potential conflict of interest.

## Footnotes

1 http://www.ncbi.nlm.nih.gov/genome/viruses/

2 www.ncbi.nlm.nih.gov/orffinder
