# Supplementary material for: Robust Virome Profiling and Whole Genome Reconstruction of Viruses and Viroids Enabled by Use of Available mRNA and sRNA-Seq Datasets in Grapevine (Vitis vinifera L.)
Source: Front Microbiol. 2020 Jun 5;11:1232. doi: 10.3389/fmicb.2020.01232 (PMC7289960; doi:10.3389/fmicb.2020.01232)
Supplement: Supplementary file 2 [file Data_Sheet_2.docx]

**Supplementary Figures (S1-S13) for the manuscript entitled "Robust virome profiling and whole genome reconstruction of viruses and viroids enabled by use of available mRNA and sRNAseq datasets in grapevine (Vitis vinifera L.)"**


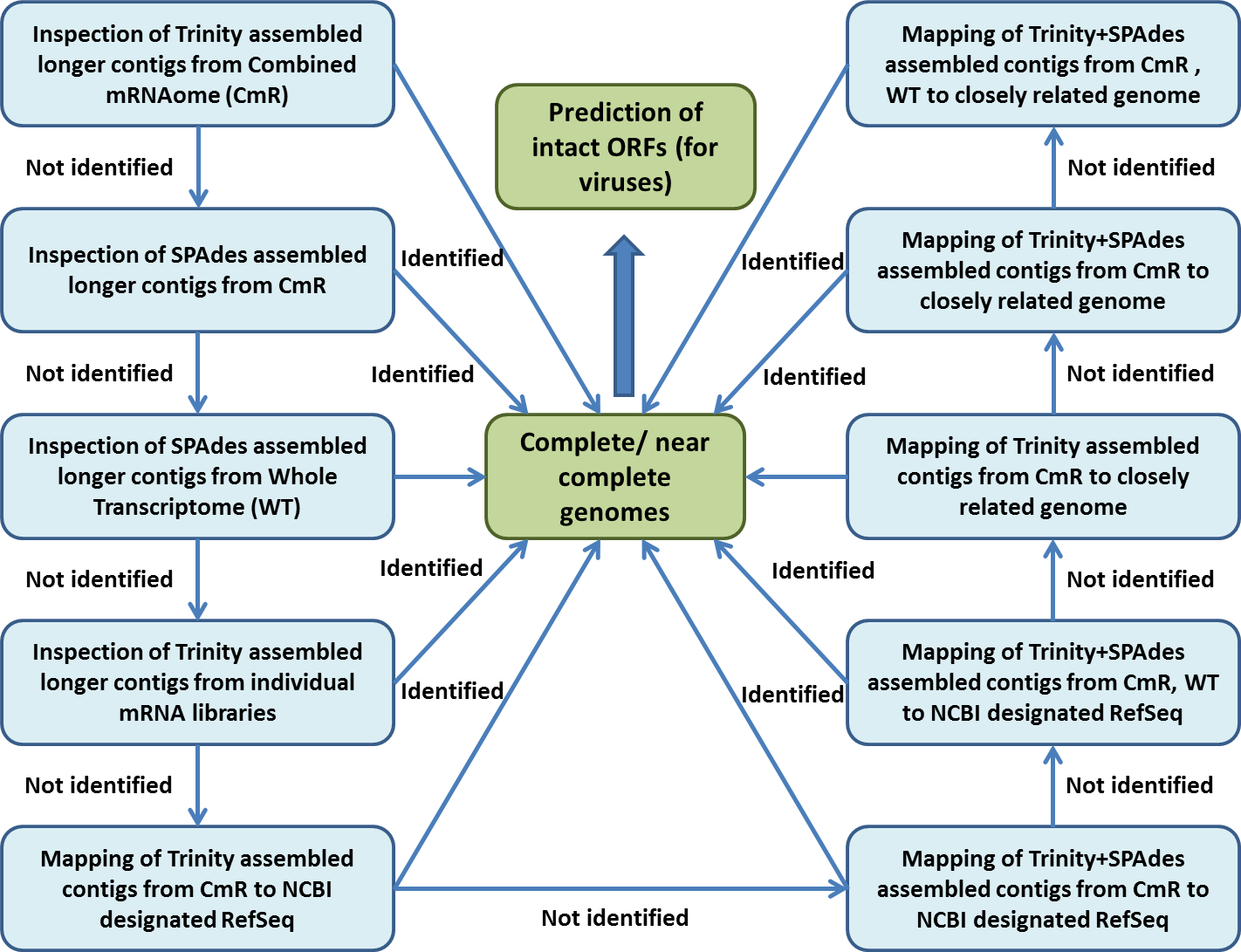


**Figure S1.** Schematic representation of various steps followed for complete/ near complete genome reconstruction. CmR- Combined mRNAome; WT- Whole Transcriptome; RefSeq- Reference Sequence designated by NCBI.


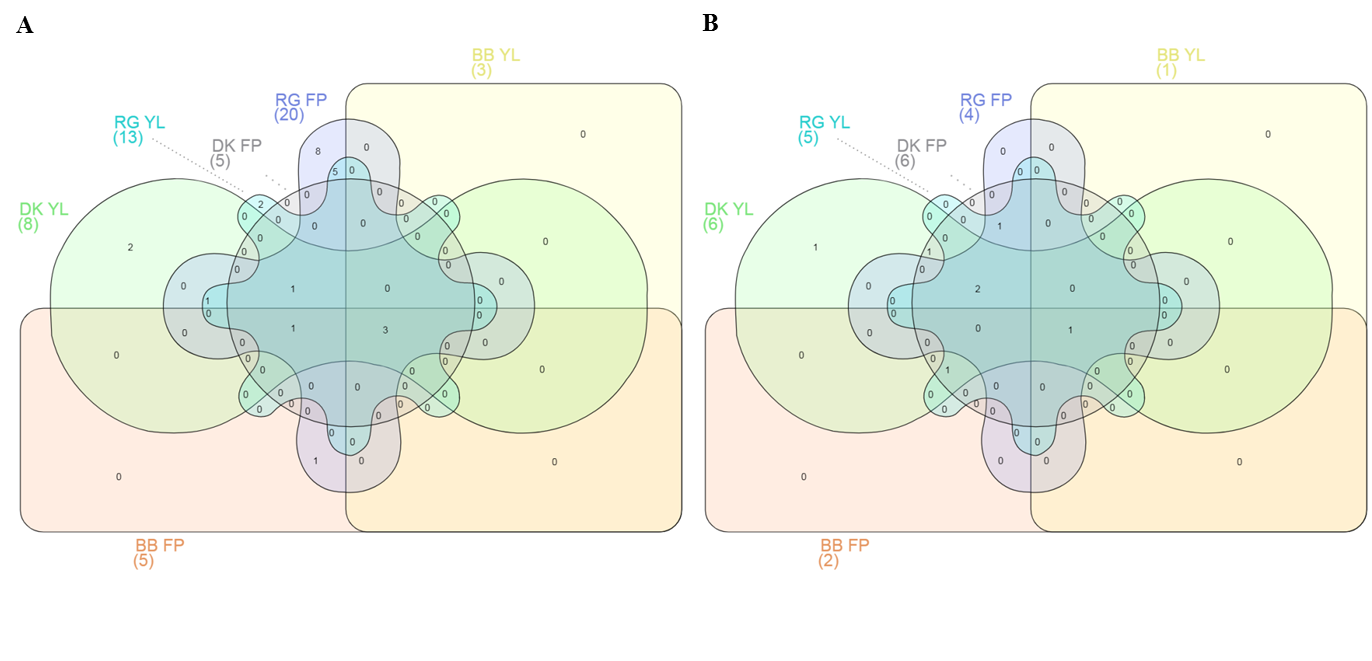


**Figure S2.** Venn diagram displaying identified viruses and viroids in two tissues (FP, YL) based on contigs assembled from individual mRNA **(A)** and sRNA libraries of each cultivar **(B)**. Venn diagrams were created using a web based tool InteractiVenn.


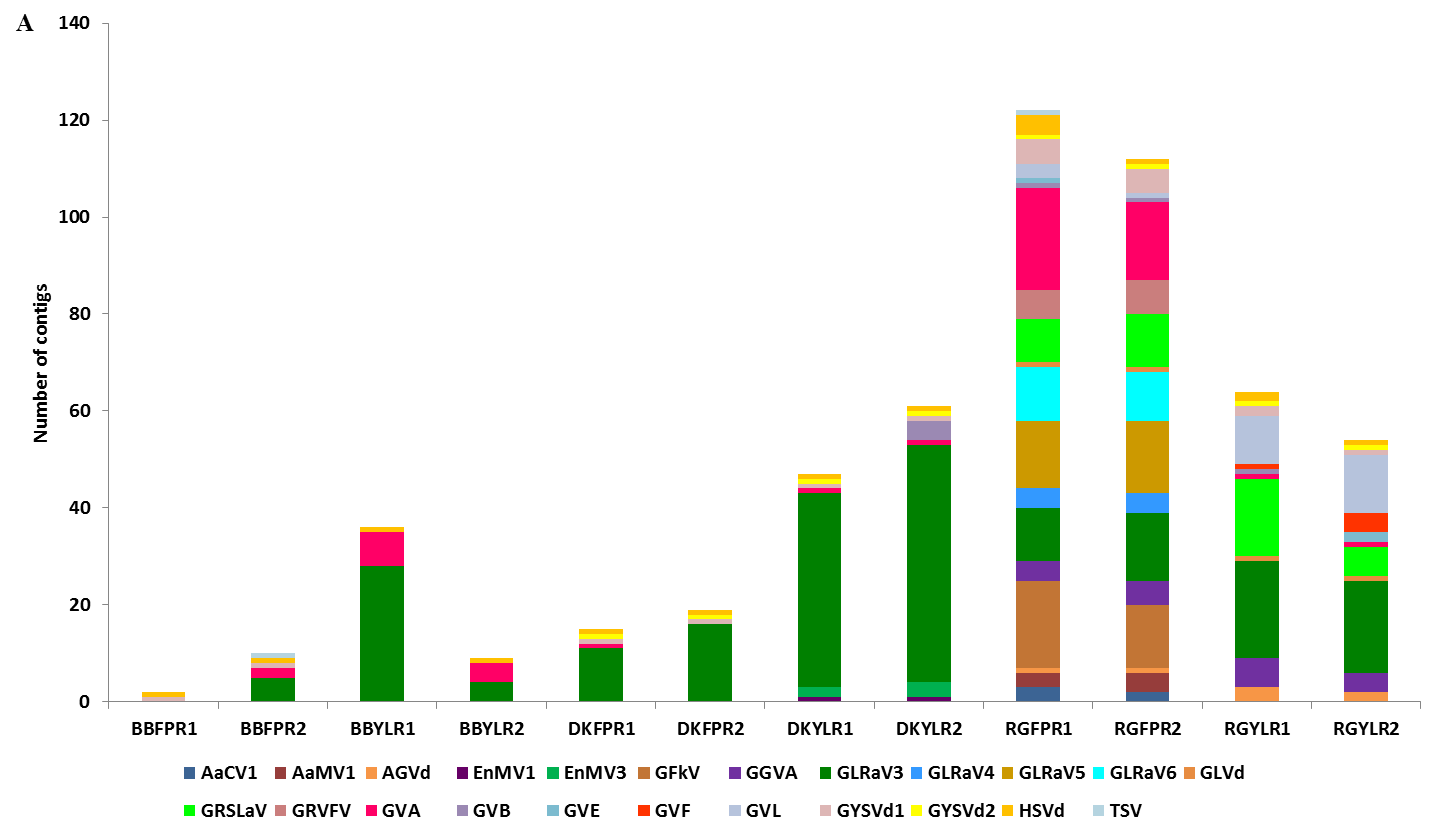

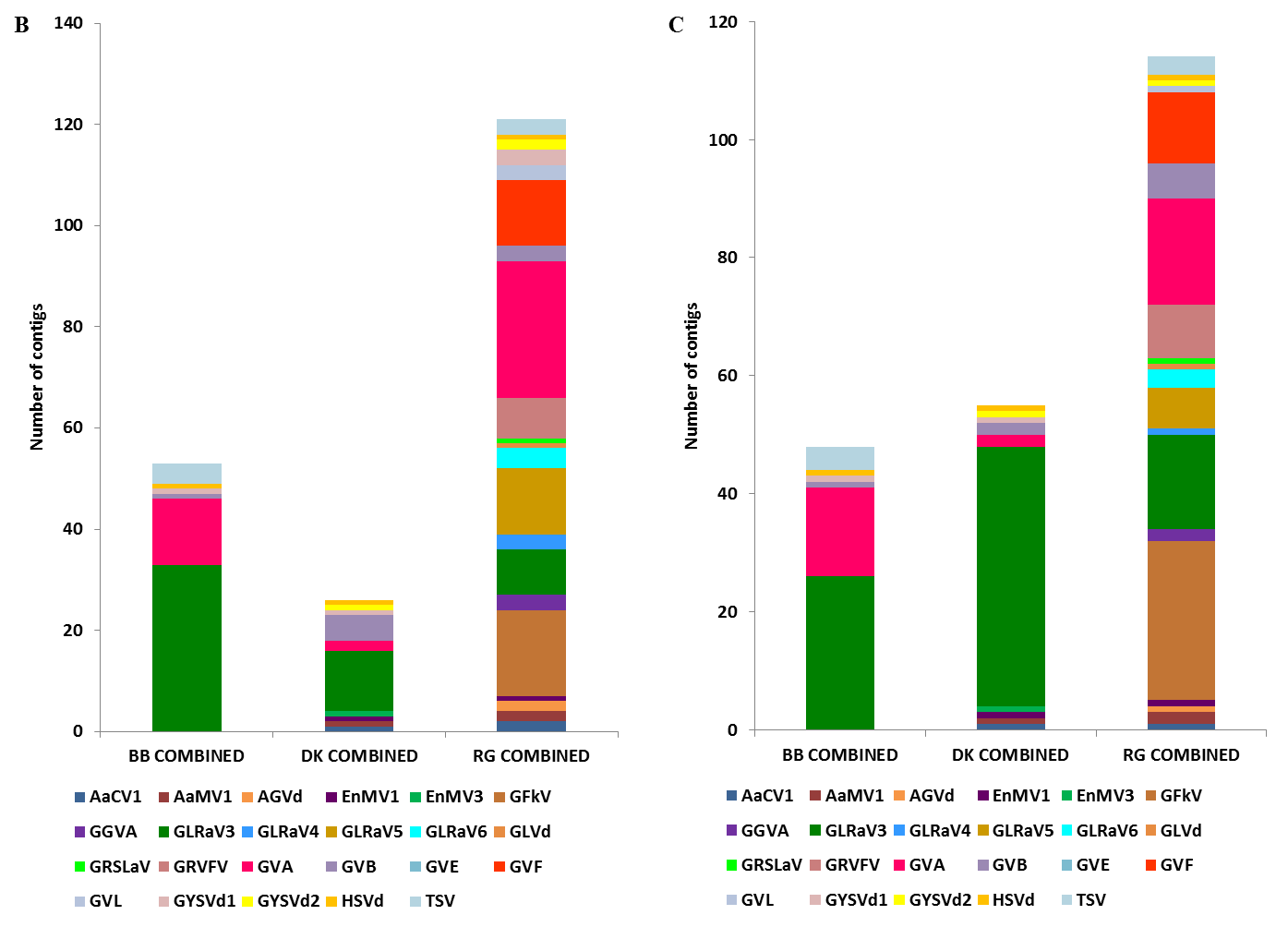


**Figure S3.** Number of viral/ viroid associated contigs assembled using Trinity in each mRNA library **(A)**, Trinity **(B)** and SPAdes **(C)** from combined mRNAome of each cultivar. Each identified virus and viroid is indicated by a different colour.


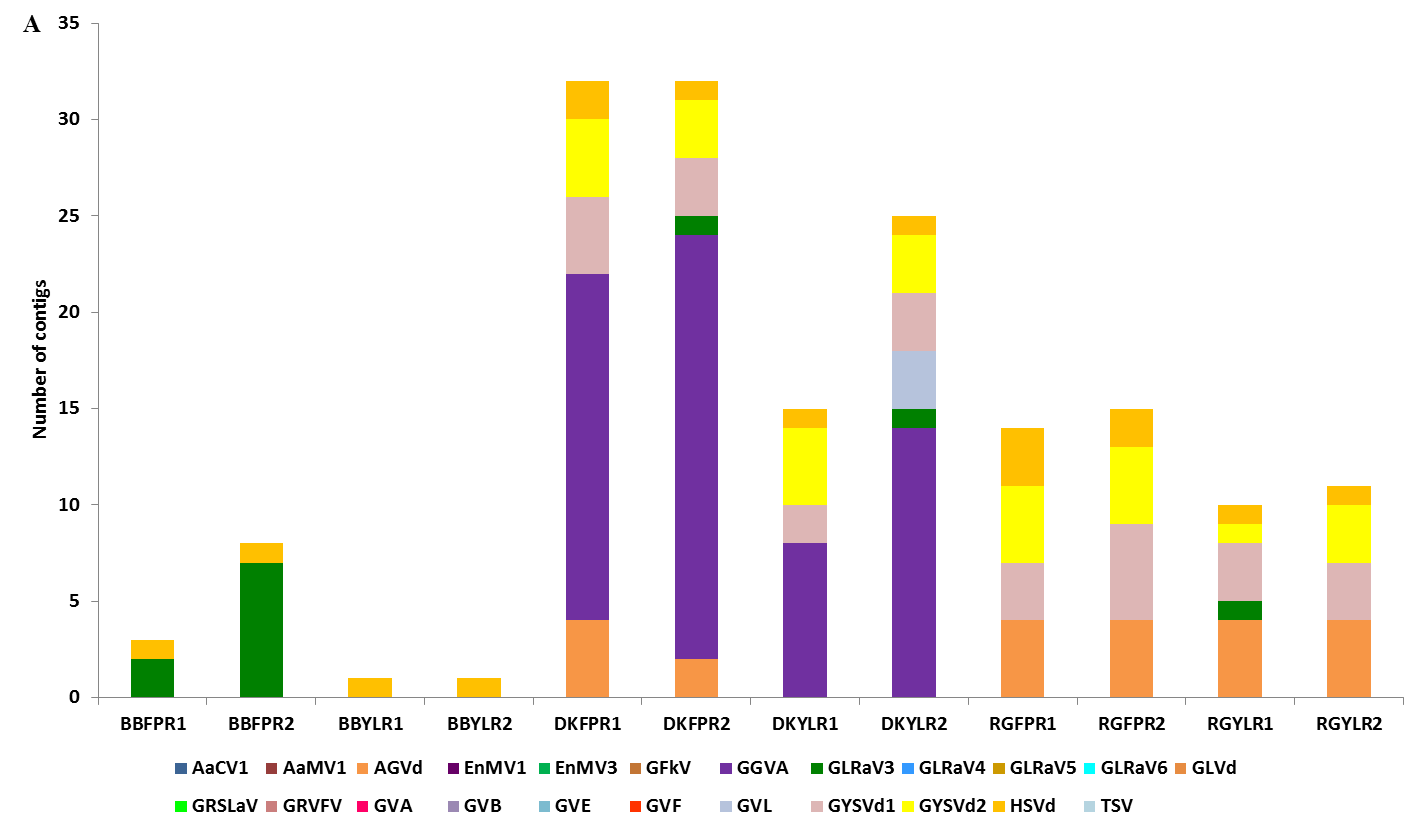

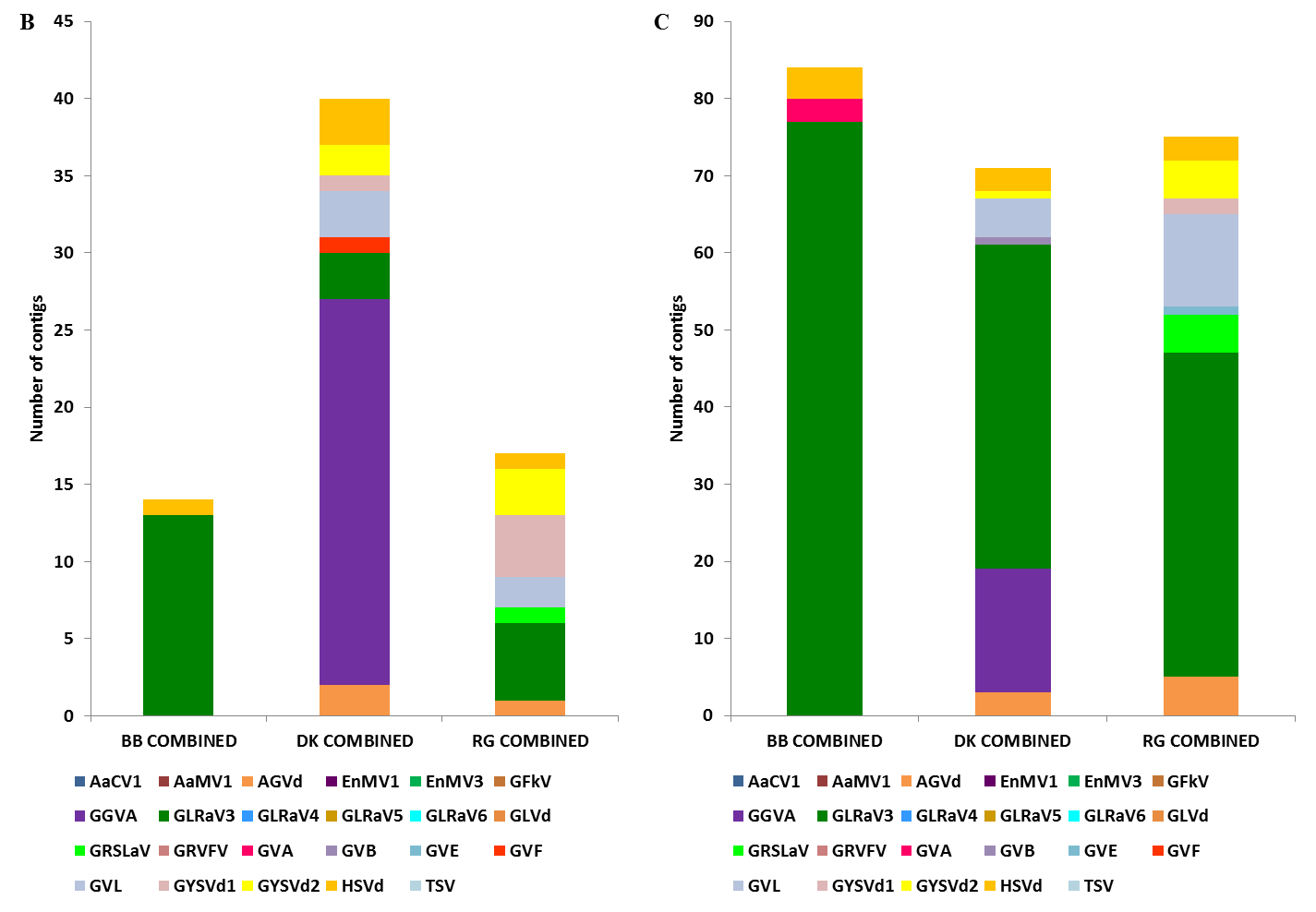


**Figure S4.** Number of viral/ viroid associated contigs assembled using CLC in each sRNA library **(A)**, CLC **(B)** and Velvet **(C)** from combined sRNAome of each cultivar. Each identified virus and viroid is indicated by a different colour.


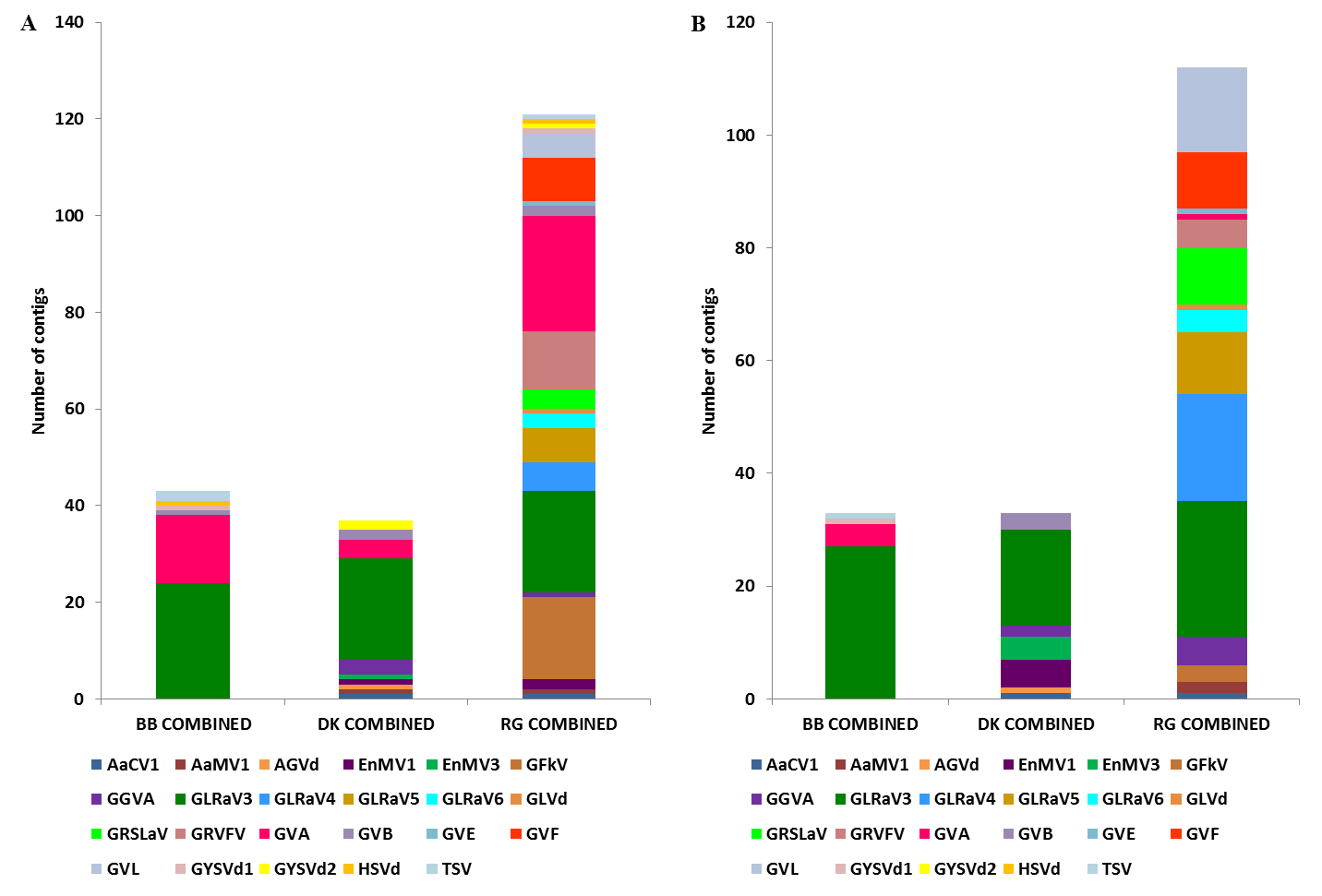


**Figure S5.** Number of viral/viroid associated contigs assembled using SPAdes **(A)** and Velvet **(B)** from whole transcriptome of each cultivar. Each identified virus and viroid is indicated by a different colour.


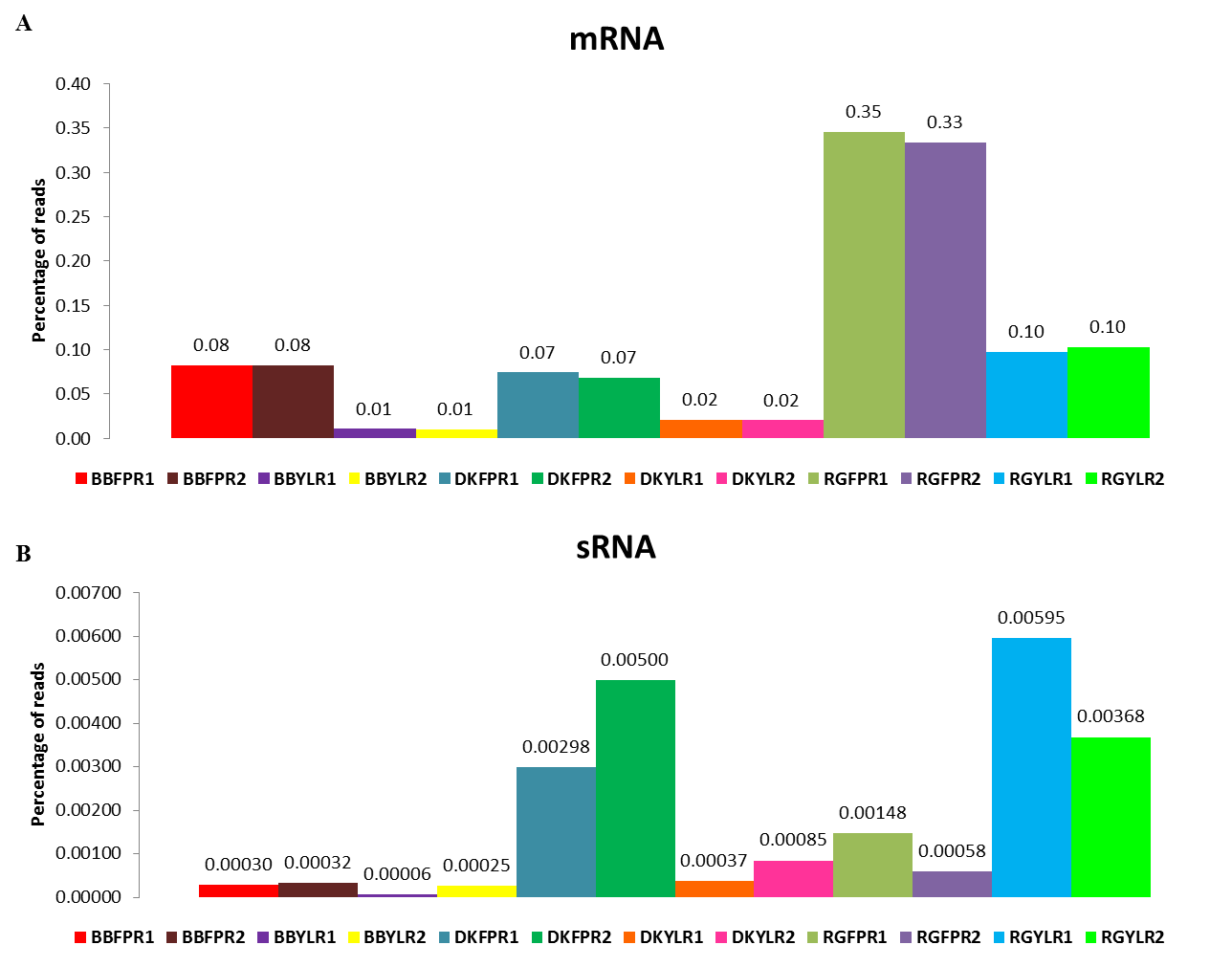


**Figure S6.** Proportion of virus and viroid associated reads in each mRNA **(A)** and sRNA **(B)** library


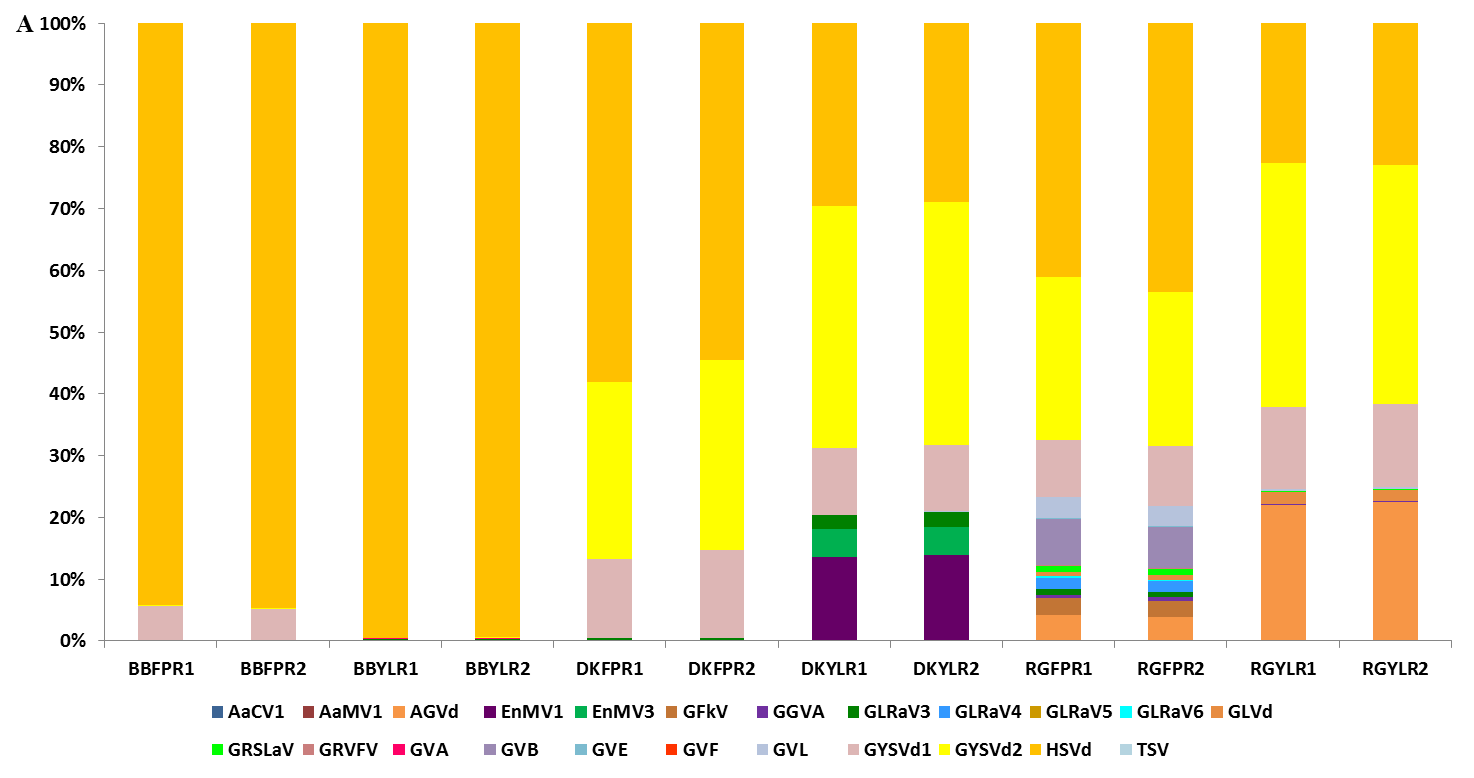


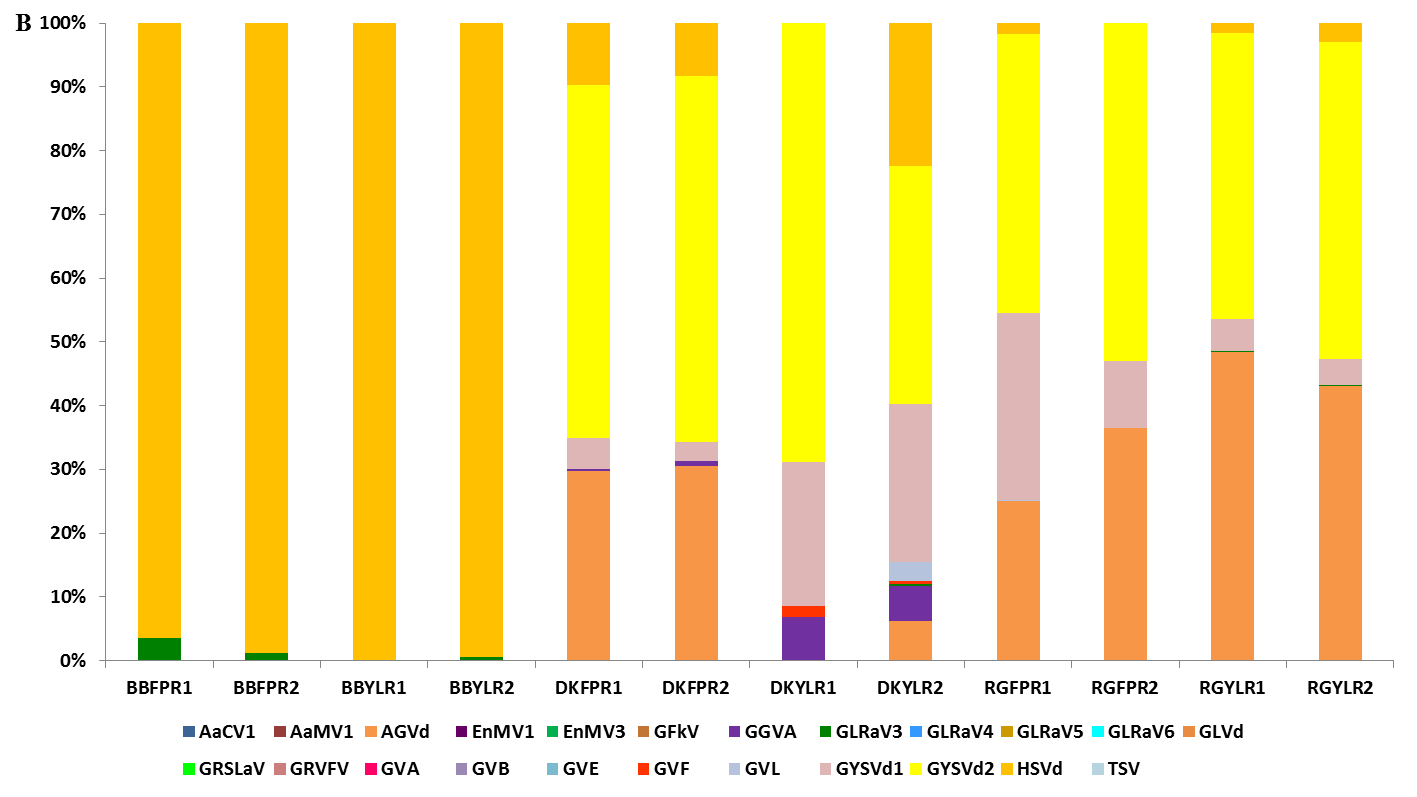


**Figure S7. Copy number estimation of viruses/ viroids identified from mRNA (A) and sRNA (B) libraries.** Each virus and viroid is indicated by a different colour.


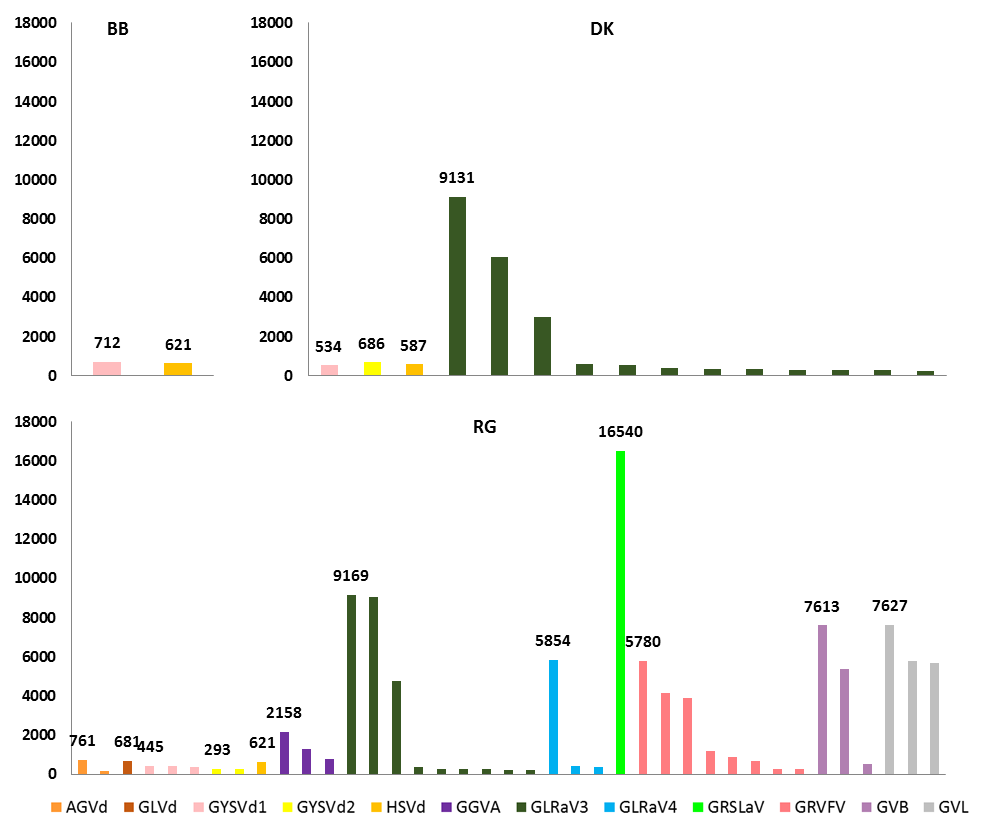


**Figure S8.** Length distribution of genome reconstructed viral and viroidal contigs obtained through combined mRNAome assembly using Trinity in three grapevine cultivars.


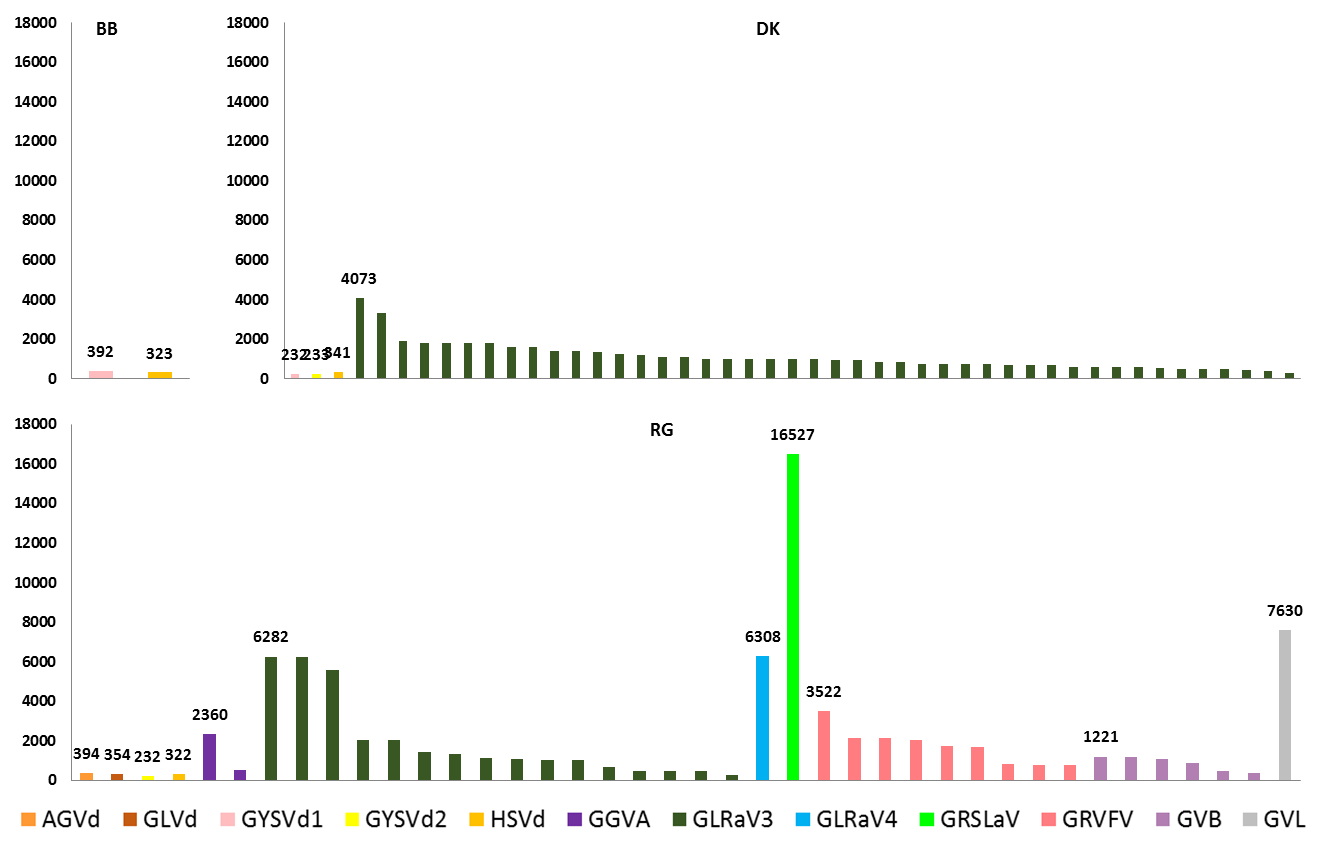


**Figure S9.** Length distribution of genome reconstructed viral and viroidal contigs obtained through combined mRNAome assembly using SPAdes in three grapevine cultivars.


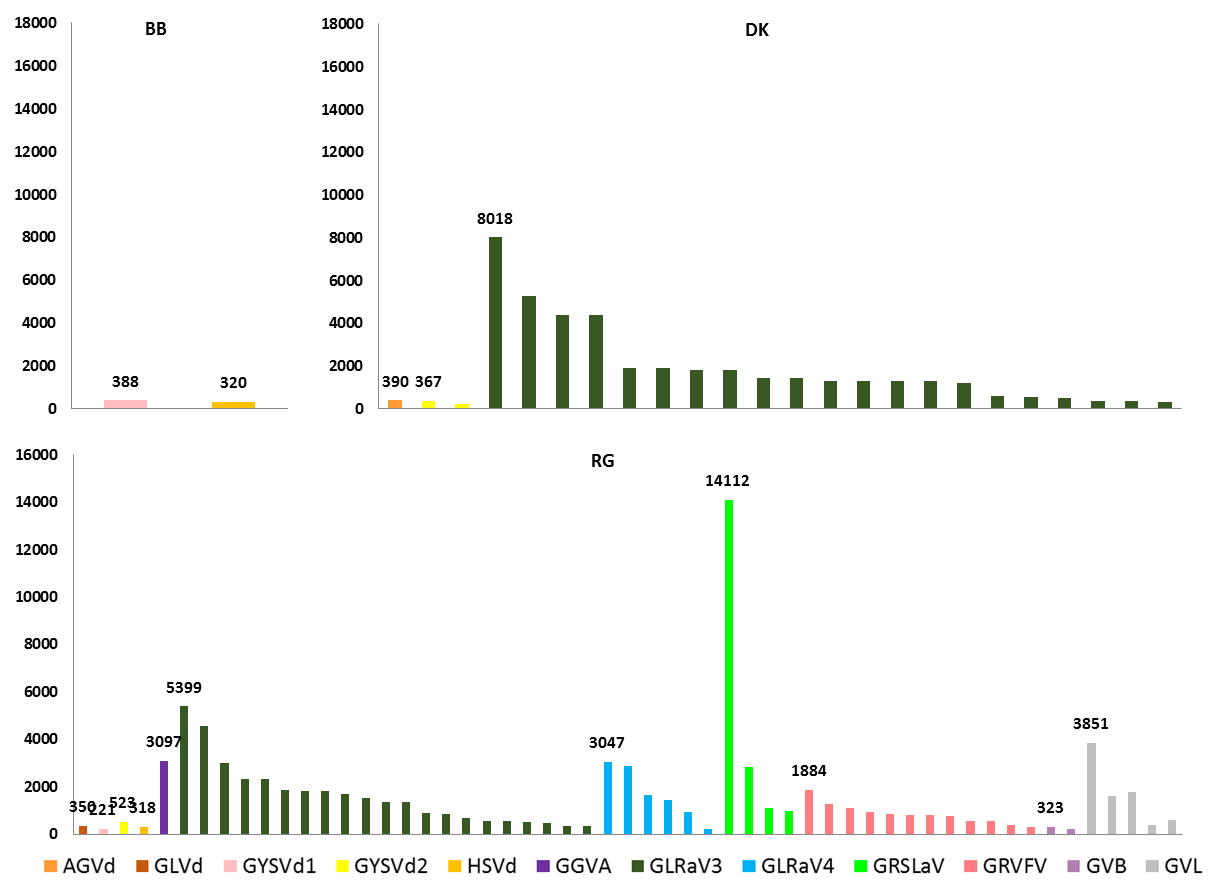


**Figure S10.** Length distribution of genome reconstructed viral and viroidal contigs obtained through whole transcriptome assembly using SPAdes in three grapevine cultivars.


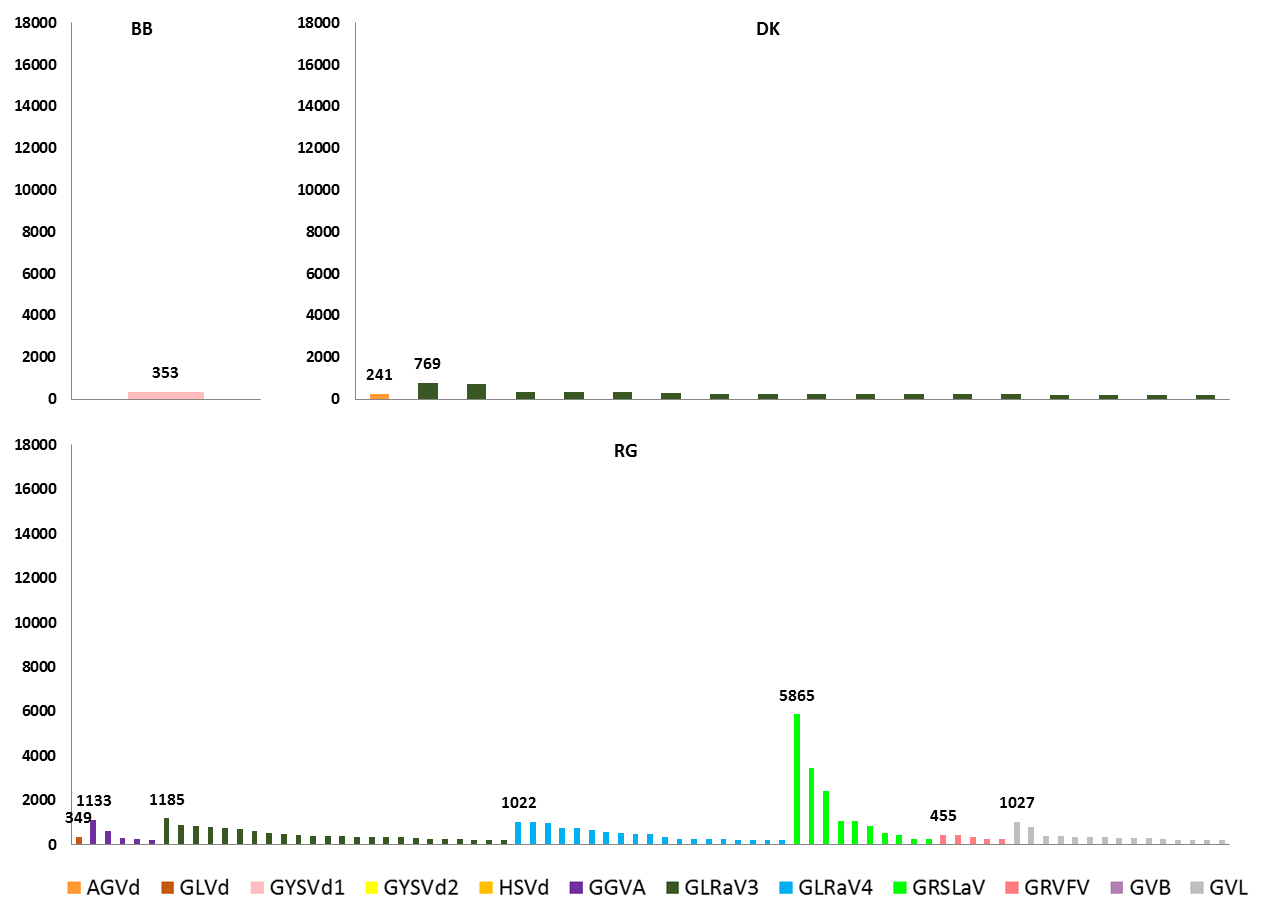


**Figure S11.** Length distribution of genome reconstructed viral and viroidal contigs obtained through whole transcriptome assembly using Velvet in three grapevine cultivars.


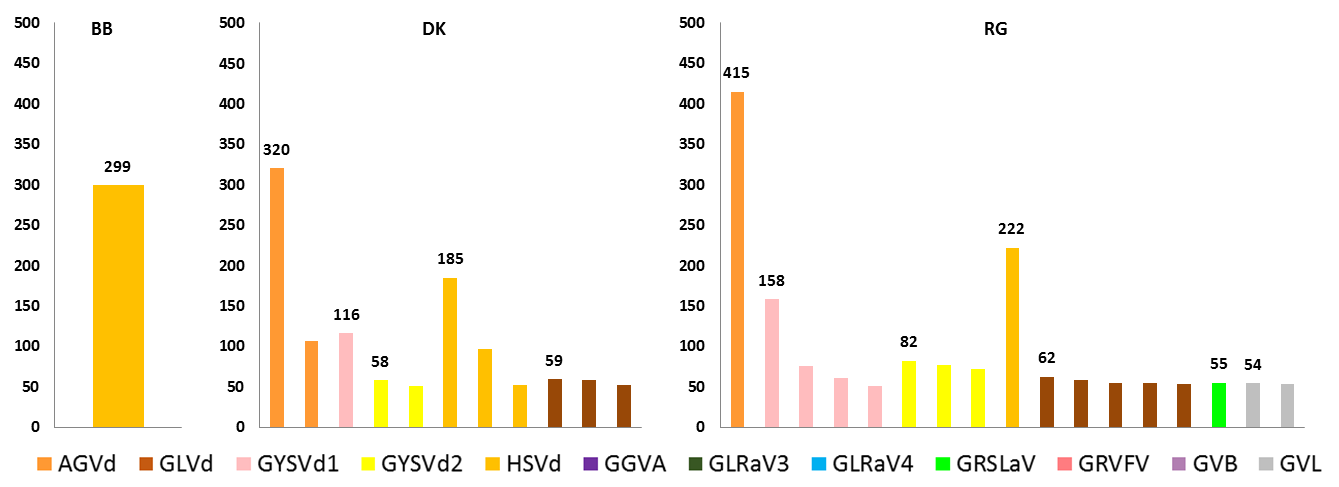


**Figure S12.** Length distribution of genome reconstructed viral and viroidal contigs obtained through combined sRNAome assembly using CLC in three grapevine cultivars.


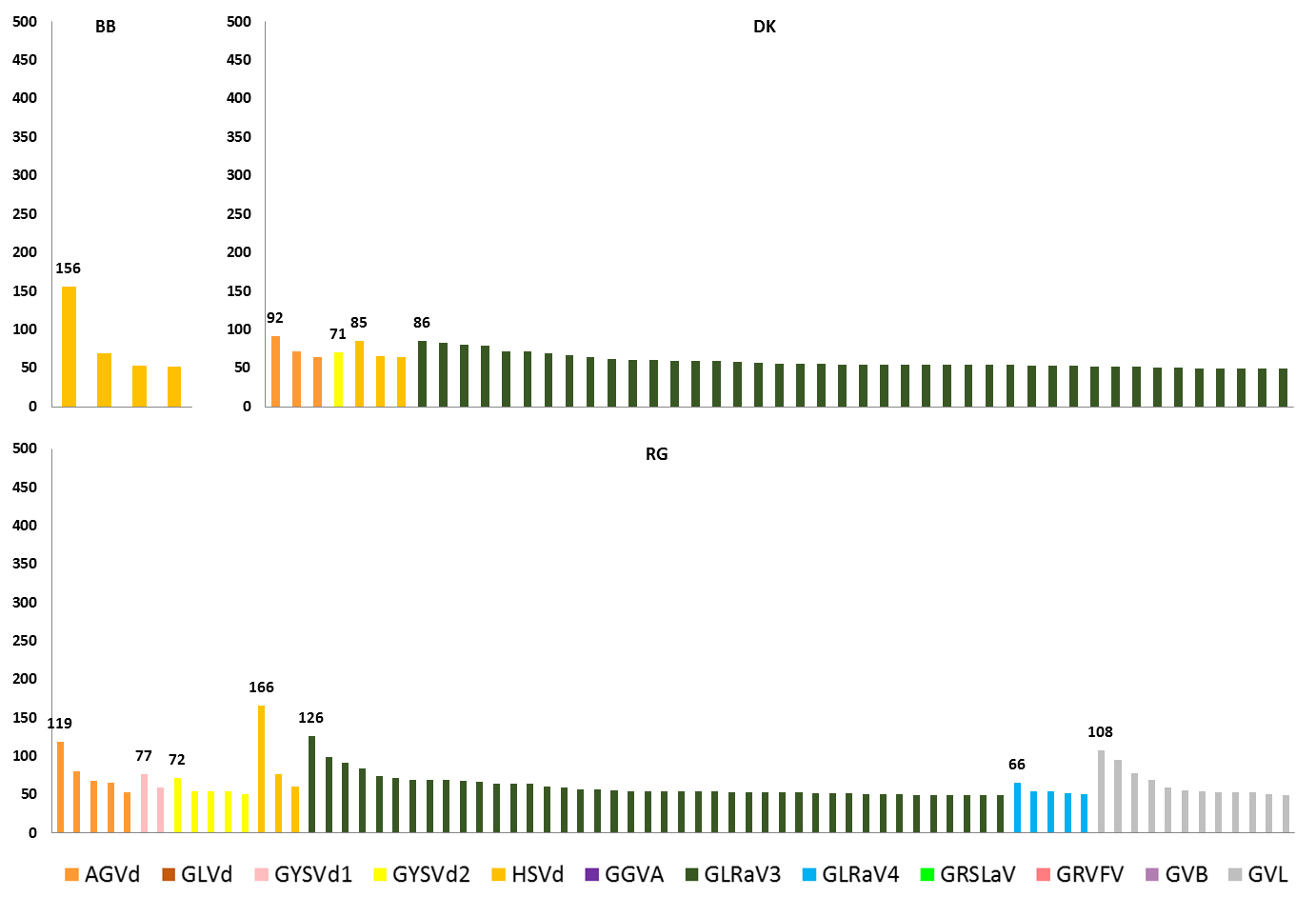


**Figure S13.** Length distribution of genome reconstructed viral and viroidal contigs obtained through combined sRNAome assembly using Velvet in three grapevine cultivars.
